# Supplementary material for: Content-rich biological network constructed by mining PubMed abstracts
Source: BMC Bioinformatics. 2004 Oct 8;5:147. doi: 10.1186/1471-2105-5-147 (PMC528731; doi:10.1186/1471-2105-5-147)
Supplement: Additional File 5 — The original Chilibot query results of the term "long-term potentiation (LTP)" and 22 other terms, limiting the latest references analyzed to the years 1990, 1995, 2000, and 2004. [file 1471-2105-5-147-S5.bz2 › chilibotAdditionalFile5/ltp2000/html/TAU_SYNAPTOPHYSIN.html]

 


 **TAU** and **SYNAPTOPHYSIN** 
  
Found 55 abstracts in PubMed,  **30 abstracts were retrieved and analyzed**.  


---

 Search Google  |
 PDF files only 
|  EDU domain only 

---

**Interactive relationship** (e.g. stimulation, inhibition, etc)

- Changes in L1 immunolabeling in the deafferented dentate gyrus corresponded in a spatial and temporal manner to changes of the synaptic marker  **synaptophysin**  and axonal marker phosphorylated  **tau** .  Ref: 8951867 Neuroscience, 1996

**Parallel relationship** (e.g. studied together, co-existance, homology, etc.)

- Marker analyses show that a member of the N CAM family 5B4 CAM,  **synaptophysin** , and especially GAP 43 and non phosphorylated  **tau** , are enriched in the GCP fraction.  Ref: 8922671 Brain Res Dev Brain Res, 1996
- However, TIs did not react with any of the following anti neurofilament protein Ab, anti  **tau**  Ab, anti paired helical filament Ab, anti tubulin Abs alpha and beta, anti neuron specific enolase Ab, anti glial fibrillary acidic protein Ab, anti  **synaptophysin**  Ab, anti myelin basic protein Ab, anti actin Ab and anti phosphorylated epitope of neurofilaments Ab.  Ref: 8866433 J Neurol Sci, 1996
- Brain derived neurotrophic factor induces a rapid upregulation of  **synaptophysin**  and  **tau**  proteins via the neurotrophin receptor TrkB in rat cerebellar granule cells.We have examined the effects of neurotrophins brain derived neurotrophic factor and nerve growth factor on the expression of the maturation specific proteins  **synaptophysin**  and  **tau** , and the growth associated protein 43 in cerebellar granule cells.  Ref: 9185679 Neurosci Lett, 1997
- In this study we analyse the hippocampal expression patterns of hyperphosphorylated  **tau** , paired helical filament PHF related protein, beta amyloid and  **synaptophysin**  in a group of Alzheimer s disease AD sufferers with and without additional pathology.  Ref: 10912925 Acta Neuropathol (Berl), 2000
- We have asked whether the expression of neuronal markers microtubule associated proteins  **tau** , MAP 2,  **synaptophysin**  p38, and the growth associated protein GAP 43 are dependent on cortical afferents or spontaneous activity.  Ref: 8861721 Brain Res Dev Brain Res, 1996
- In contrast, the hypoglossal motor nucleus displayed immunoreactivity from 1 PN for  **synaptophysin** , synaptotagmin, SNAP 25, GAP 43,  **tau** , and MAP 2.  Ref: 8725306 J Comp Neurol, 1996
- In this study, we have found that, during the first 10 postnatal days 1 10 PN, the facial motor nucleus lacked immunoreactivity for  **synaptophysin** , synaptotagmin, GAP 43,  **tau** , and SNAP 25.  Ref: 8725306 J Comp Neurol, 1996
- NT2 cells in these grafts acquired molecular markers of fully mature neurons including the low, middle, and high molecular weight neurofilament proteins, microtubule associated protein 2,  **tau** , and  **synaptophysin** .  Ref: 8978473 J Comp Neurol, 1996
- These neurons displayed extensive processes, exhibited immunoreactivity for mature neuron specific markers such as  **tau**  and  **synaptophysin** , and fired action potentials upon current injection.  Ref: 10679769 J Neurosci Res, 2000
- After 10 PN, immunoreactivity increased in the facial motor nucleus for  **synaptophysin** , synaptotagmin, GAP 43, and  **tau** .  Ref: 8725306 J Comp Neurol, 1996
- In addition to the routine examination including methenamine Bodian staining, an immunohistochemical study was performed, using antibodies to amyloid beta protein,  **tau** , ubiquitin, glial fibrillary acidic protein GFAP,  **synaptophysin** , and Ki MIP a marker of the microglial and perivascular cells.  Ref: 9600680 J Neurol Sci, 1998
- METHODS We have studied, by immunohistochemistry and immunoblotting, the presence of neuronal cytoskeletal antigens class III beta tubulin isotype beta III, microtubule associated proteins MAP2, MAP1B and  **tau** , and different neurofilament NF protein components, and  **synaptophysin**  in comparison with the glial fibrillary acidic protein GFAP and S 100 protein in the PC12 cell line.  Ref: 9517852 Anat Rec, 1998
- Immunohistochemical studies showed positive staining for neurofilament protein NF, neuron specific enolase NSE,  **synaptophysin** , MAP 2,  **tau** , NCAM 180, vimentin, and S 100 protein.  Ref: 8971690 Mol Chem NeuropatholMol Chem Neuropathol, 1998
- The developmentally controlled expression of skeletal muscle specific genes coding for myf5, myogenin, myoD and myf6, alpha 1 subunit of the L type calcium channel, cell adhesion molecule M cadherin, and neuron specific genes encoding the 6 60, and 200 kDa neurofilament proteins, synaptic vesicle protein  **synaptophysin** , brain specific proteoglycan neurocan, and microtubule associated protein  **tau**  was demonstrated by RT PCR analysis.  Ref: 9521839 Exp Cell Res, 1998
- There were several subsets of neurites that were argyrophilic or immunoreactive against antibodies to either phosphorylated  **tau**  protein, phosphorylated neurofilaments, ubiquitin, or  **synaptophysin** .  Ref: 10378375 Acta Neuropathol (Berl), 1999
- **Synaptophysin** , neurofilaments and  **tau**  labeling was found in cells forming the pineocytomatous rosettes.  Ref: 9600600 Acta Neuropathol (Berl), 1998
- Double labeling studies showed that all three of these neuropathological structures also contained ubiquitin,  **synaptophysin** , and neurofilament but not  **tau**  immunoreactivity.  Ref: 9466562 Am J Pathol, 1998
- However, at 39 degrees C, the cells produced dendritic, neuronal like processes and elevated levels of NF68 and MAP2, as well as the neuronal markers  **synaptophysin** , neurone specific enolase, and low levels of  **tau** , all determined by western blotting and immunofluorescent staining.  Ref: 10537044 J Neurochem, 1999
- Compared with other neuronal markers, the positive ratio of alpha synuclein was not as high as  **synaptophysin** , microtubule associated protein 2, neuron specific enolase and  **tau** , but it was higher than neurofilament and chromogranin A.  Ref: 10672322 Acta Neuropathol (Berl), 2000
- However, the IIBs did not react with any of the following anti neurofilament protein antibodies Abs 6 60 and 200 kDa, anti neuron specific enolase antibody Ab, anti  **tau**  Ab, anti tubulin Abs alpha and beta, anti paired helical filament Ab, anti actin Ab, anti phosphorylated epitope of neurofilaments Ab, anti  **synaptophysin**  Ab, anti myelin basic protein Ab, anti actin Ab and anti glial fibrillary acidic protein Ab.  Ref: 9129116 J Neurol Sci, 1997
- On immunstaining some of the OLCs were positive for  **synaptophysin**  and negative for glial fibrillary acidic protein GFAP, glucocerebroside,  **tau**  and MAP 2.  Ref: 9832257 Clin NeuropatholClin Neuropathol, 1997
- We find that BDNF but not NGF rapidly upregulates levels of  **synaptophysin** ,  **tau**  and c Fos correlating with expression of the neurotrophin receptor TrkB.  Ref: 9185679 Neurosci Lett, 1997
- Unlike the epithelial parental Panc 1 cells, these cells exhibited the appearance of a neuroendocrine like phenotype with extensive branch like processes, and marked cytoplasmic and cytoskeletal immunostaining for  **tau**  2,  **synaptophysin** , and chromogranin A.  Ref: 9569027 Oncogene, 1998
- The neuronal phenotype of PC12 cells was confirmed by staining for MAP2,  **tau** , and NF proteins, as well as for  **synaptophysin** .  Ref: 9517852 Anat Rec, 1998
- The clustered pericapillary bodies were immunostained by antibodies to phosphorylated neurofilament,  **synaptophysin** , and ubiquitin...  **tau**  protein, and paired helical filaments.  Ref: 8841662 Acta Neuropathol (Berl), 1996
- Dystrophic neurites were not detected by a modified Bielschowsky stain or by neurofilament,  **synaptophysin**  and  **tau**  immunohistochemistry in the olfactory mucosa.  Ref: 9004244 Neuropathol Appl Neurobiol, 1996
